# Evolving Towards Mutualism

**DOI:** 10.1371/journal.pbio.1000279

**Published:** 2010-01-12

**Authors:** Diana Gitig

**Affiliations:** Freelance Science Writer, White Pains, New York, United States of America

**Figure pbio-1000279-g001:**
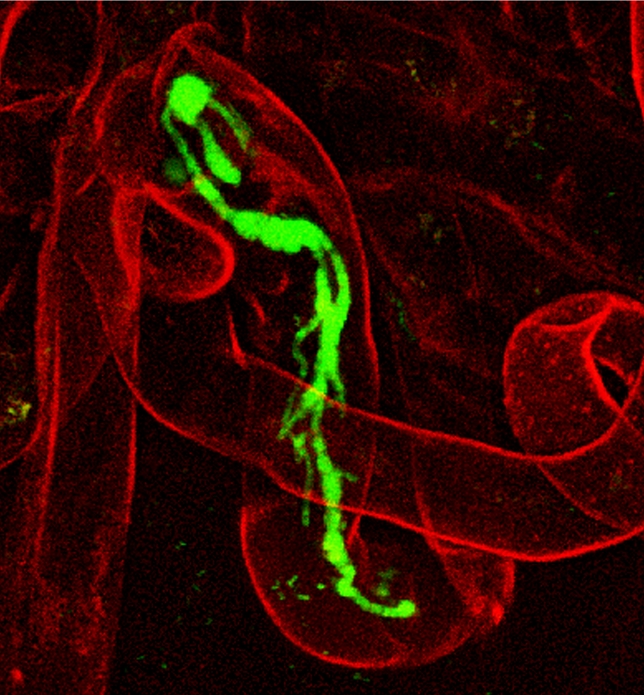
Infection thread formed by symbiotically evolved *Ralstonia solanacearum* in root hairs of the legume *Mimosa pudica*. (Image credit: Ton Timmers).

[Fig pbio-1000279-g001]Plants, and all other living things, require nitrogen for growth; it is an essential component of nucleic acids and proteins. Although air is mostly nitrogen, this gaseous form is inaccessible to plants and must be fixed into ammonium to render it biologically relevant. Soil bacteria called rhizobia fix nitrogen, but to do this they must first take up residence inside the roots of legumes like pea, alfalfa, clover, and soybean.

Soon after a legume begins to grow, rhizobia invade its root hairs and multiply, causing the plant to form specialized organs—nodules—that contain the proliferating bacteria. This symbiotic arrangement benefits both parties: legumes can thrive without nitrogen fertilizers only if they have functional nitrogen-fixing nodules, while the bacteria receive the energy needed to multiply and fix nitrogen from the plant. When the plant dies, the fixed nitrogen is released into the soil so other plants can use it. This process has significant implications for agriculture, as nitrogen is the most common nutrient deficient in the earth's soil and, thus, the one most commonly supplied by chemical fertilizers.

Rhizobia are a diverse group taxonomically, genetically, and metabolically. They can be found in distant genera. Their symbiotic trait appears to have arisen independently multiple times by horizontal transfer of genes. However, it is not thought that this horizontal gene transfer is sufficient to confer symbiosis, or to explain the biodiversity of rhizobia. There must be selective pressures preventing or permitting the expression of the acquired symbiosis trait and adaptive mechanisms to deal with these pressures. But neither the pressures nor the measures taken to circumvent them are known.

As described in this issue of *PLoS Biology*, Catherine Masson-Boivin and her colleagues used experimental evolution to study what converts a pathogenic bacterium into a rhizobium. To begin with, the researchers transferred a plasmid conferring the symbiosis trait from a nitrogen-fixing symbiont into a pathogenic strain of bacteria. The resultant strain could not nodulate the host, so they took advantage of the legume's ability to select for nodulation-proficient mutants in a heterogeneous population and used bacterial whole-genome resequencing to identify two types of mutations required for mutualism. One type inactivated the type III protein secretory system (T3SS), a well-known pathogenic determinant, allowing nodulation and early infection. Another adaptive mutation in a virulence master regulatory gene additionally allowed for full intracellular invasion of nodule cells, but the nodules degenerated prematurely, only three weeks after inoculation.

Although the mutant strain did not achieve mutualism, the results are informative. The finding that one master regulator is so important in the transition from pathogenesis to symbiosis fits nicely with the idea that such global regulators are preferred evolutionary targets. And the fact that the evolved clones expressed different degrees of the symbiotic trait indicated that other stages in the process, such as rhizosphere colonization, host specificity, and of course nitrogen fixation, might one day be demystified with similar methodologies.

This study not only shows that the adaptation of the bacterial genome to selective pressure from a legume is essential to the evolution and diversification of rhizobia, but also provides a means for identifying further effectors and processes involved in the establishment of symbiosis.

**Marchetti M, Capela D, Glew M, Cruveiller S, Chane-Woon-Ming B, et al. (2010) Experimental Evolution of a Plant Pathogen into a Legume Symbiont. doi: 10.1371/journal.pbio.1000280**

